# Polymeric Materials for Rare Earth Elements Recovery

**DOI:** 10.3390/gels9100775

**Published:** 2023-09-24

**Authors:** Hongtao Zhang, Yongfeng Gao

**Affiliations:** 1 School of Chemistry and Chemical Engineering, Qinghai Normal University, Xining 810008, China; zhanght21@lzu.edu.cn; 2 Department of Chemistry, University of Alberta, Edmonton, AB T6G 2G2, Canada

**Keywords:** rare earth elements (REEs), polymeric materials, polymeric resins, polymer membranes, cross-linked polymer networks, nanocomposite polymers

## Abstract

Rare earth elements (REEs) play indispensable roles in various advanced technologies, from electronics to renewable energy. However, the heavy global REEs supply and the environmental impact of traditional mining practices have spurred the search for sustainable REEs recovery methods. Polymeric materials have emerged as promising candidates due to their selective adsorption capabilities, versatility, scalability, and regenerability. This paper provides an extensive overview of polymeric materials for REEs recovery, including polymeric resins, polymer membranes, cross-linked polymer networks, and nanocomposite polymers. Each category is examined for its advantages, challenges, and notable developments. Furthermore, we highlight the potential of polymeric materials to contribute to eco-friendly and efficient REEs recovery, while acknowledging the need to address challenges such as selectivity, stability, and scalability. The research in this field actively seeks innovative solutions to reduce reliance on hazardous chemicals and minimize waste generation. As the demand for REEs continues to rise, the development of sustainable REEs recovery technologies remains a critical area of investigation, with the collaboration between researchers and industry experts driving progress in this evolving field.

## 1. Introduction

Rare earth elements (REEs) constitute a group of 17 chemically akin elements, located in the middle of the periodic table (atomic numbers 21, 39, and 57–71), playing pivotal roles in both established and cutting-edge industries like lighting [1], electronics [2], renewable energy [3,4], and aerospace [5]. They are integral components in diverse technologies such as fluorescent lamps, lasers, supermagnets, atomic batteries, and engine turbines. However, global REEs supply has been heavily reliant on China, which has provided over 85% of the world’s production since the late 20th century [4,6,7,8]. This dependency raises concerns, as REEs are often byproducts of other mining operations, making their extraction and recovery less efficient. Additionally, the accumulation of REEs in the environment are a potential harm to living organisms and pose environmental and health risks [9]. Consequently, there is a growing need for sustainable and eco-friendly REEs recovery methods. Numerous technologies, including solvent extraction [10,11,12], ion exchange [13,14], precipitation [15], crystallization [16], and adsorption [17,18,19], have been employed to recover REEs from various sources, such as ores, industrial waste streams, and environmental samples. These methods are designed to selectively extract and separate REEs, offering potential solutions to the challenges associated with their supply and environmental impact.

Solvent extraction, a widely adopted approach, entails mixing an organic solvent, typically kerosene or a similar hydrophobic substance [10], with an aqueous solution containing REEs. It is based on the principle that different elements or ions can be selectively extracted from a liquid phase into an organic solvent phase owing to differences in their chemical properties, such as solubility and affinity for certain extracting agents. Complexing agents or extractants are introduced into the solvent to preferentially bind to REEs. The selective extraction and separation of REEs from other elements can be achieved by manipulating factors like pH and various conditions [20,21]. Solvent extraction has the advantages of achieving high selectivity for specific REEs, scalable for both laboratory and industrial applications, and extracting agents can be tailored for different REEs. However, the drawback is the complex processes involved, and environmental and safety considerations [22].

Ion exchange is renowned for its remarkable selectivity and efficiency in REE recovery. This method employs ion exchange resins that are impregnated with specific ions tailored to capture REEs as the solution flows through them [23]. Both the pH of the aqueous solution and the flow rate can affect the ion exchange reactions. Ion exchange can be highly selective for specific REEs, depending on the choice of resin and operating conditions. However, the regeneration of ion exchange resins and the impurities are still challenges. Subsequent elution using an appropriate solution allows for the retrieval of these valuable elements. Depending on the resin and elution method used, ion exchange can have operating costs associated with chemicals and resin replacement.

Another technique involves the selective precipitation of REEs from a solution by adjusting pH levels and introducing specific precipitating agents [24]. This method is commonly employed to initially concentrate REEs from dilute solutions before subjecting them to further purification processes. It involves the chemical conversion of dissolved REE ions in a solution into solid, insoluble compounds or precipitates, which relies on the differences in solubility between REE compounds at varying conditions, such as pH and temperature. Precipitation can be tailored to selectively recover specific REEs based on the chemical properties, and this method is relatively straightforward and suitable for various REE-bearing materials. However, impurity co-precipitation, and regeneration are still challenges for this method.

Crystallization processes can also be utilized for REEs recovery [25]. In this approach, a suitable compound is crystallized, and this compound is subsequently decomposed to isolate the individual REEs. This method takes advantage of differences in solubility between REE compounds under varying conditions. It has the advantages of high purity, selectivity, and scalability. Thus, crystallization has been used in primary mining operation and recycling processes to recover REEs from various sources. However, this method sees less frequent use due to its intricate and complex nature. 

To selectively adsorb REEs from a solution, a variety of adsorbents, including both polymeric materials [26] and inorganic compounds [27], can be employed. Functionalized polymers or ligands are often incorporated into these adsorbents to enhance selectivity. The recovery of rare earth elements encompasses a diverse range of techniques, each characterized by its own unique advantages and complexities. These methods continue to evolve as researchers endeavor to develop more efficient, selective, and environmentally friendly approaches to extract and utilize REEs.

Each of these methods has its advantages and disadvantages, depending on factors such as the composition of the source material, the desired purity of the recovered REEs, and environmental considerations. Often, a combination of methods is used in a multistep process to achieve high-purity REEs recovery. The choice of methods depends on the specific circumstances and goals of the REEs recovery process [28,29,30]. Over the past few decades, there has been a noteworthy surge in interest regarding the use of polymeric materials for the recovery of REEs. In this review, we will classify polymeric materials into several distinct categories, encompassing polymeric resins, polymer membranes, cross-linked polymer networks, and nanocomposite polymers. We will include illustrative examples within each category, highlighting their exceptional performance in recovering Rare Earth Elements (REEs). The provided case studies vividly showcase the remarkable adsorption capacities and the regenerable characteristics exhibited by these materials. 

## 2. Polymeric Materials for REEs Recovery

Polymeric materials have recently gained considerable attention due to their potential applications in the retrieval of REEs [31,32,33,34,35]. These materials offer a suite of advantages, including their capacity for selective adsorption, adaptability for functionalization, scalability, regenerability, minimized environmental footprint, and waste reduction.

One notable advantage lies in their ability to be fine-tuned for high selectivity toward specific REEs. By embedding functional groups within the polymer matrix [36], these materials can be precisely engineered to preferentially adsorb particular REEs, streamlining the separation processes. Furthermore, polymeric materials can be easily customized through functionalization, permitting the incorporation of specific ligands or groups that enhance their affinity for rare earth elements [37]. This augmentation significantly improves the efficiency of both adsorption and recovery processes. Versatility is another hallmark, as polymeric materials can be crafted into diverse forms such as beads, fibers, membranes, and films [38,39,40]. This adaptability renders them suitable for various processing scales, spanning from laboratory experimentation to large-scale industrial implementation.

In terms of sustainability, many polymeric materials employed for REEs recovery exhibit regenerable properties, allowing for multiple cycles of use. This not only conserves valuable resources but also curtails waste generation. When juxtaposed with traditional solvent-based extraction methods, polymeric materials proffer a more environmentally conscientious approach to REEs recovery. They typically operate under gentler conditions and produce fewer deleterious byproducts. Moreover, these materials facilitate the concentration of REEs from dilute sources, thereby diminishing the volume of waste necessitating management. 

Numerous categories of polymeric materials have undergone rigorous investigation to assess their viability for REEs recovery [33,35,41,42,43]. These materials hold substantial potential as a pivotal component in forthcoming REEs extraction procedures, contributing to sustainable practices and a decreased environmental footprint. In the following sections, we will introduce various types of polymeric materials that have been employed in studies related to the recovery of REEs.

### 2.1. Polymeric Resins

Polymeric resins possess remarkable adaptability, as they can be precisely customized through functionalization to exhibit a strong preference for specific REEs [44,45,46]. Their utility extends beyond selectivity to include advantages like ease of regeneration, scalability for various operational scales, and a reduced environmental impact when compared to the more traditional solvent-based extraction methods. Extensive research endeavors in this field have yielded substantial advancements and enriched insights into the application of polymeric resins for the efficient recovery of REEs [46,47,48].

**Figure 1:**
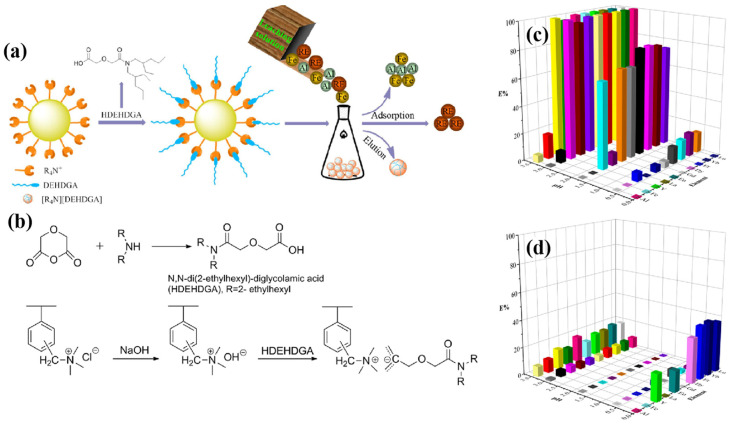
(**a**) Illustration of polymer resin used for REEs recovery. (**b**) A schematic of the preparation of *N*,*N*-di(2-ethylhexyl)-diglycolamide grafted polymer resin. Relationship among element, pH, and adsorption capacities for (**c**) [R4N][DEHDGA] resin and (**d**) the DGA−normal resin. Reprinted with the permission from Ref. [49].

Polymeric resins can be modified with various functional groups to selectively adsorb specific REEs from complex mixtures [49,50]. The choice of resin and functional groups is crucial in achieving high selectivity. A system based on the *N*,*N*-di(2-ethylhexyl)-diglycolamide (DEHDGA) grafted polymer resin, used for the efficient separation of RE(III), is proposed by Wang et al. [49] Upon grafting of DEHDGA ligand, the resulting polymer resin was evaluated for its RE(III) adsorption performance. Utilizing a multifaceted approach involving pH-dependent adsorption, kinetics, and equilibrium adsorption isotherm experiments, the study findings illuminate the paramount role of pH in shaping the adsorption characteristics of the resin for RE(III). Consequently, a comprehensive competitive adsorption model is proposed, which effectively accounts for the concurrent adsorption of RE(III) and protons. One of the pivotal outcomes of this investigation is the revelation of the resin’s heightened selectivity towards RE(III) when subjected to a pH environment of 3.0, particularly in the presence of Al(III) and Fe(III). This remarkable selectivity contrasted favorably with the performance of the conventional diglycolamides(DGA)-normal resin, as vividly depicted in Figure 1. Moreover, the outcomes of batch adsorption experiments unequivocally underscore the substantial enhancement in adsorption capacities for RE(III) achieved through the application of this resin. This enhancement stands in stark contrast to the performance of the DGA-normal resin, reaffirming the exceptional potential of the prepared resin in bolstering the capture of RE(III) species. 

**Figure 2:**
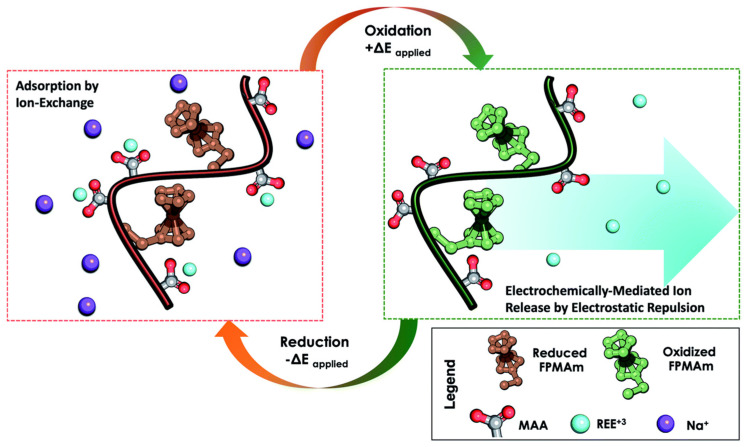
Overview of the reversible capture and release of REEs by P(FPMAm−co−MAA) through electrochemically regenerated ion exchange. During adsorption, REE ions are captured by chemical ion exchange. During desorption, ferrocene (Fc) is oxidized to ferrocenium (Fc^+^) electrochemically, allowing for desorption of the REE ions through electrostatic repulsion. Reduction of ferrocenium (Fc^+^) back to ferrocene (Fc) is required for electrode cycling. Reprinted with the permission from Ref. [42].

Polymeric resins offer a cost-effective and sustainable solution for REEs recovery due to their capability for multiple eco-friendly regeneration cycles. Su and collaborators [42] have introduced an innovative strategy for recovering REEs employing a redox-copolymer named poly(ferrocenylpropyl methacrylamide-co-methacrylic acid) (P(FPMAm-co-MAA)). This remarkable copolymer combines ion-exchange carboxyl groups for REEs adsorption with a redox-active ferrocene component for regeneration, all under precise electrochemical control. By molecularly tuning the copolymer composition, efficient adsorption uptake could be achieved alongside electrochemically regenerated adsorbent reuse without the use of additional chemicals, as shown in Figure 2. Redox-copolymers containing carboxylic acid and ferrocene moieties were synthesized and studied for their effectiveness in recovery of REEs (Y, Nd, Eu, Gd, Dy, and Ce) from aqueous solutions. The copolymer showed increasing REE adsorption capacities with increasing content of MAA (the REEs binding group), with a 50/50 ratio of ferrocenyl groups to carboxylic acid groups providing an optimal balance between uptake and electrochemical regeneration. Adsorption of Y(iii) on P1-CNT showed an equilibrium capacity of 69.4 mg Y(iii) per g polymer at an optimal pH of 6. Electrochemical desorption of the adsorbed REE from the electrodes was achieved using a positive potential vs. Ag/AgCl, to release the bound cation by electrostatic repulsion without the need for additional stripping reagents, achieving close to full regeneration under electrochemical conditions. The adsorption capacity of the electrodes remained relatively constant during four consecutive cycles, confirming the structural stability of the redox-active copolymer.

Polymeric resins bring numerous advantages to the table when it comes to recovering REEs. Researchers have diligently explored the intricacies of REEs adsorption kinetics and thermodynamics on polymeric resins, offering invaluable insights into the factors that shape adsorption efficiency [45]. Furthermore, extensive experimentation with continuous column setups has illuminated the practicality of deploying polymeric resins at an industrial scale, emphasizing their real-world potential. Concurrently, a concerted drive towards more environmentally conscientious REEs recovery processes using polymeric resins has emerged [51,52]. The focus here is on reducing the reliance on hazardous chemicals and curbing the generation of waste.

In essence, the utilization of polymeric resins for REEs recovery holds great promise. Ongoing research is geared towards refining these methods for heightened efficiency and sustainability. As the demand for REEs continues its upward trajectory, the development of innovative, eco-friendly REEs recovery technologies remains a critical domain of investigation. Collaboration between researchers and industry experts actively addresses the challenges and opportunities within this evolving field.

### 2.2. Polymer Membranes

Membranes showcase a distinctive ability to selectively transport specific ions while excluding others, rendering them a strategic choice for the separation of REEs from complex mixtures [7,13,53,54,55]. Tailoring membranes for precise selectivity enables the exact separation of REEs. In contrast to conventional methods, membrane processes provide superior REEs selectivity and decreased energy consumption, aligning with environmentally sustainable practices [56,57,58]. Furthermore, these processes hold the potential for achieving zero liquid discharge. Nevertheless, a critical aspect revolves around evaluating the economic viability of employing membrane techniques in REEs recovery, demanding meticulous consideration. Membrane separation processes offer a promising pathway for extracting REEs from waste effluents, offering numerous benefits. These encompass elevated recovery rates, exceptional selectivity, minimal space requirements, and reduced sludge generation. Various types of membranes, such as nanofiltration (NF) [59,60,61], ultrafiltration (UF) [62,63,64], and reverse osmosis membranes [65], find application in these processes. In evaluating composite polymeric membranes with various functionalities, we conducted a comparative analysis of commonly utilized polymeric membranes in the context of REE recovery, as summarized in Table 1.

**Figure 3:**
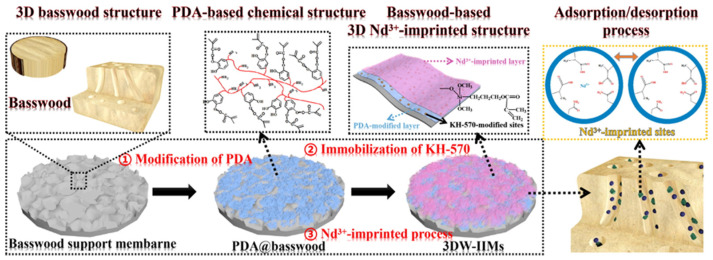
Schematic diagram for the synthesis procedures of 3DW-IIMs. Reprinted with the permission from Ref. [66].

To gain a deeper insight into the functionality of polymer membranes in REEs recovery, we will use the study conducted by Pan and colleagues as an illustrative example. A two-step composite modification and imprinting technique was devised and executed, employing rare-earth Nd(III) as the template ions [66]. Illustrated in Figure 3, this process began with a bioinspired self-assembly nanocomposite approach that introduced the polydopamine(PDA)-based membrane reaction platform. Subsequently, an innovation-driven development phase was implemented before the Nd(III)-imprinted polymerization, involving the immobilization of polymerizable double bonds on PDA@basswood. To enhance the selectivity and separation performance of our synthesis method, a two-step-temperature imprinting method was refined, utilizing MAA and AM as the dual functional monomers. Following a straightforward removal procedure of Nd(III) from the prepared membrane, specific recognition sites for Nd(III) ions were ultimately attained.

Yan and his team engineered ion-imprinted electrospun membranes (Y-IIEMs) meticulously designed for the selective separation of the closely-related heavy rare earth ion Y(III) from Ho(III) and Er(III) by applying high efficient ligand-inducing and aqueous phase ion-imprinting [67]. Cyanex272 served a dual purpose as both a carrier in the membrane matrix and a highly efficient ionic ligand within the imprinted layer. Through PDA bionic adhesion and sol-gel aqueous phase imprinting, Cyanex272’s thermal stability saw substantial improvement. The impressive water contact angle of Y-IIEMs, measuring only 23.2°, was likely the result of a synergistic combination of polar groups, including P=O and P-OH from the Cyanex272 ligand, imino groups from the pDA layer, and silicon hydroxyls from the sol-gel imprinting process.

**Figure 4:**
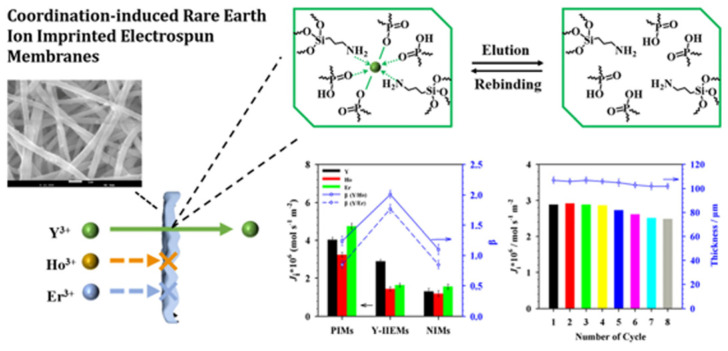
Schematic illustration of IIM preparation for specific recognition of REEs. Reprinted with the permission from Ref. [67].

Furthermore, the calculated static adsorption imprinting factor for Y(III) exceeded 7, affirming the successful formation of valid recognition sites. During dynamic permeation, the separation factors β(Y/Ho) and β(Y/Er) exhibited significant enhancements, increasing from 1.24 and 0.85 for non-imprinted membranes to 2.01 and 1.77 for imprinted ones. Impressively, after undergoing 8 cycles of dynamic permeation, Y-IIEMs only experienced a minor 13.8% reduction in flux, highlighting their remarkable stability and reusability, as shown in Figure 4.

Polymer inclusion membranes (PIMs) represent a vital category within membrane separation technologies [13,53,68,69]. These membranes are characterized by a 3D-polymer framework, utilizing materials like polyvinylidene difluoride (PVDF), polytetrafluorethylene (PTFE), or cellulose triacetate (CTA), to embed ion-complexing carriers. What sets PIMs apart is their capacity to maintain flexibility during operational conditions, achieved simply by introducing a plasticizer or carefully selecting the appropriate polymer material. This selection accounts for factors like molecular weight, morphology, as well as bulk and surface chemistry. PIMs offer numerous advantages over non-polymer alternatives, a field still relatively underexplored in the scientific literature. These advantages encompass their straightforward composition, exceptional versatility (tailored for various types of REEs solutes with a single membrane), ease of synthesis scalable for tunable porosity, effective carrier immobilization during manufacturing, robust mechanical properties, enduring operational stability, rapid and convenient installation in facilities, and, importantly, cost-efficiency.

Although there are many benefits for using polymer membranes in REEs recovery, they also come with challenges related to several issues that require careful consideration and ongoing research to overcome. For example, setting up membrane-based systems can have higher initial capital cost compared to some conventional methods. Fouling, caused by the accumulation of impurities on the membrane surface, can reduce membrane efficiency over time, necessitating cleaning and maintenance. 

### 2.3. Cross-Linked Polymer Network

A cross-linked polymer network, often referred to simply as a cross-linked polymer, is a three-dimensional structure formed by chemically linking polymer chains together [70,71,72]. This type of polymer network is characterized by strong covalent bonds between individual polymer chains, creating a mesh-like structure. Cross-linked polymer networks are essential materials in many industries due to their exceptional strength, durability, and resistance to chemical and thermal influences. They play a crucial role in the development of high-performance materials for diverse applications.

Inorganic adsorbents, once widely used, fell out of favor due to their limitations, including poor selectivity and environmental instability. Consequently, there has been a shift towards the adoption of more specialized functional polymeric adsorbents [73]. Following extensive research, polymeric adsorbents featuring amidoxime, phosphate, amide, and carboxyl groups have emerged as promising candidates, exhibiting impressive adsorption capacities for uranium and REEs [74].

Among these, amidoxime-based adsorbents [75] have demonstrated exceptional uranium adsorption capabilities, owing to their strong attraction to uranyl ions. However, a notable challenge in their use stems from the complex preparation processes. These processes typically involve the utilization of highly toxic, explosive, and volatile acrylonitrile monomers. Moreover, the need for labor-intensive amidoximation and alkali treatments prior to adsorption can present obstacles to scalability and hinder sustainable development efforts.

Hydrogels, recognized as porous cross-linked polymer networks, have emerged as highly effective sorbents for REEs [76,77,78,79]. These hydrophilic copolymers, although typically not soluble in water, feature expansive and flexible chelating groups thoughtfully positioned within their three-dimensional structures [80,81,82]. Significantly, the incorporation of inorganic fillers has shown immense promise in augmenting the performance of hydrogel networks [83]. The amalgamation of organic and inorganic elements in hybrid gels has been a game-changer, mitigating the limitations often associated with traditional hydrogels. These enhancements facilitate the efficient recovery of REEs [84].

**Figure 5:**
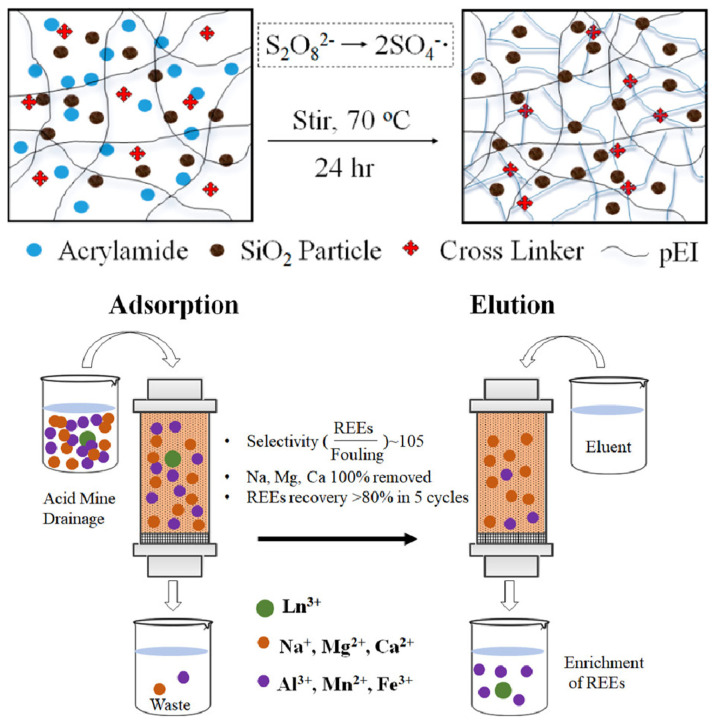
Preparation of Grafted Hybrid Hydrogel Network for adsorption of rare-earth metal. Reprinted with the permission from ref. [77].

Gray and his research team meticulously crafted organic-inorganic hybrid gels composed of polyethylenimine(PEI)-polyacrylamide(pAAm)-SiO_2_. They conducted a comprehensive study, probing the adsorption characteristics of these gels concerning REEs metals under various solution conditions [77]. The researchers conducted an in-depth exploration into the influence of multiple reaction conditions, comprising cross-link degree, grafting degree, and SiO_2_ concentration, along with the examination of testing conditions like contact time and pH, on the efficiency of adsorption. They meticulously developed a comprehensive adsorptive mechanism to explain these phenomena. Furthermore, the reusability of the sorbent for recycling REEs was thoroughly investigated through five adsorption-desorption cycles, simulating the recovery process from a synthetic acid mine drainage solution, as shown in Figure 5. PEI-pAAm-SiO_2_ hydrogel was prepared via thermopolymerization by grafting AAm monomer onto PEI25000 as a template polymer in the presence of *N*,*N*-methylene-bis-acrylamide(MBAA) as a cross-linker at 70 °C. 

In the investigation, the organic-inorganic hybrid gel showcased remarkable adsorption capabilities. This achievement stemmed from meticulous optimization of the three-dimensional network by controlling the degree of cross-linking, grafting, and SiO_2_ concentration. A noteworthy discovery was the favorable influence of nearly neutral pH conditions on the enhanced adsorption of REEs. This observation suggests that elevating the solution pH to higher levels could serve as a practical approach to enhance REEs uptake from real-world solutions. 

**Figure 6:**
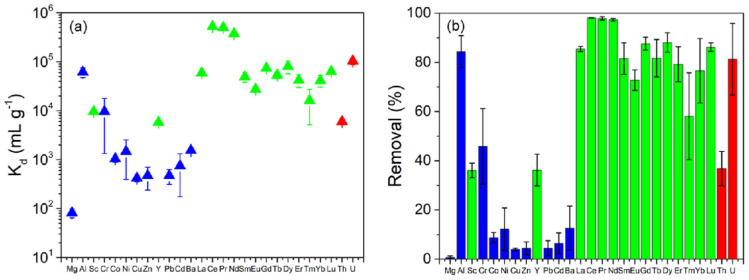
(**a**) Distribution coefficients (K_d_) of PPN for major competing ions and REE species assessed in coal fly ash leachate at pH 5.0. (**b**) Percent removal of competing ions and REE species by 100 mg L^−1^ PPN from coal fly ash leachate at pH 5.0. Elements on the X−axis are labeled in order of increasing molecular weight. Major competing ions are marked blue, REEs in green, and radionuclides in red. Reprinted with the permission from Ref. [32].

Lowry and co-workers developed a phosphate polymer nanogel (PPN) to selectively recover REEs from low REEs content waste streams [32]. As illustrated in Figure 6, the removal efficiency for most lanthanide REEs stands near or above 80%, with Ce, Pr, and Nd displaying removal rates exceeding 95% at a PPN concentration of only 100 mg L^–1^. While the removal of Sc and Y (~35%) is somewhat lower than that of the lanthanides, it still surpasses the removal rates of most competing ions such as Mg, Co, Ni, Cu, Zn, Pb, Cd, and Ba. Notably, Al remains the most formidable competing cation, exhibiting removal rates akin to some of the lanthanides. Apart from the REEs, the PPN exhibits the capability to remove approximately 80% of uranium (U) and about 30% of thorium (Th) from the leachate. This phenomenon arises from phosphate’s high affinity for radionuclides, suggesting potential applications of PPN in the realm of radioactive waste management.

**Figure 7:**
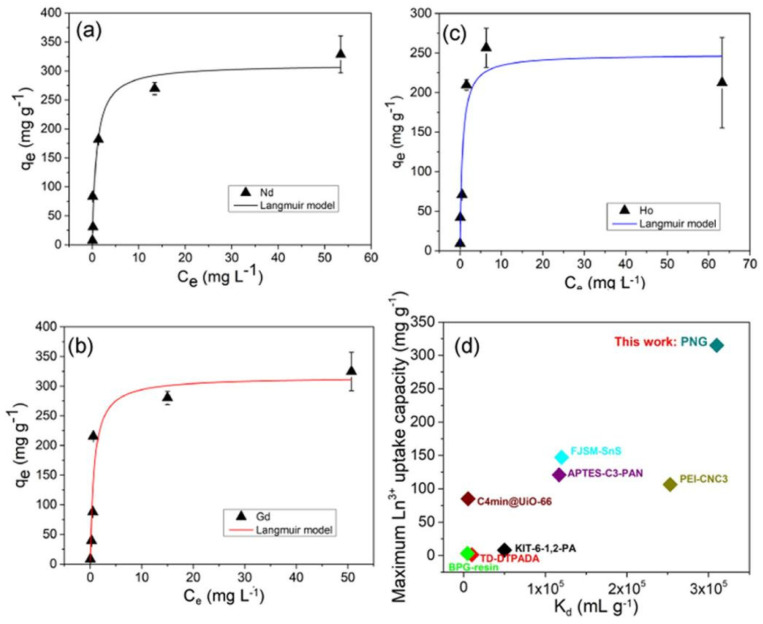
Sorption isotherm of (**a**) Nd, (**b**) Gd, and (**c**) Ho at pH 5.0 with Nd, Gd, or Ho concentration varying from 1 to 100 mg L^−1^ with 100 mg L^−1^ PPN concentration with no other species added to the solution. (**d**) Comparison of lanthanide (Ln^3+^) saturation uptake capacity (qm) and distribution coefficient (K_d_) for PPN and other benchmark REE sorbent materials at pH ≤ 7.5. Reprinted with the permission from Ref. [32].

The PPN exhibited notable sorption capacities, registering values of 311 ± 28 mg g^–1^, 316 ± 38 mg g^–1^, and 249 ± 29 mg g^–1^ for Nd, Gd, and Ho, respectively (as depicted in Figure 7a–c). This equates to a REE uptake of 1.6–2.2 mmol g^–1^. Remarkably, this sorption capacity exceeded that of several other materials. Specifically, it was nearly twice that of a layered A_2_Sn_3_S_7_·1.25H_2_O ion exchange material [85], over three times greater that of the Cr-MIL-101 MOF and TiP@GO material [86], over five times higher than a functionalized chitosan-silica hybrid material [87,88], and over ten times greater than that of ligand-functionalized silica particles and ion exchange resin (Figure 7d) [89,90,91]. Based on its chemical composition, the PPN has the potential to capture approximately 3 mmol g^–1^ of trivalent REEs if all the phosphate groups are fully deprotonated. This implies that REEs utilize up to roughly 70% of the phosphate groups within the PPN material at its maximum capacity. Despite this incomplete phosphate utilization, the REE sorption capacity of PPN remains notably high when compared to other available sorbents. Detailed comparisons for Ln^3+^ are provided in Figure 7d, where the PPN exhibited the highest maximum uptake capacity and Kd among twelve other SPE materials reported in the literature. The findings suggest that PPN has the potential to capture a substantial amount of trivalent REEs, and it outperforms numerous other reported sorbents in terms of maximum uptake capacity and Kd values.

This situation potentially opens the door to utilizing alternative sources of REEs, thereby diminishing reliance on energy-intensive and environmentally harmful REEs mining practices. The impressive capacity of PPN offers a more efficient REEs recovery process by minimizing the required number of REEs sorption/elution cycles. This reduction leads to decreased operational expenses and a smaller water footprint. While PPN exhibits good selectivity against numerous competing cations, it still removes Al^3+^ and Fe^3+^. Future research endeavors should aim to enhance selectivity, particularly concerning these crucial competing ions, particularly at low pH levels.

### 2.4. Nanocomposite Polymers

These strategies involve the integration of nanoparticles that exhibit specific affinities for rare earth elements into polymer matrices, thereby bolstering the overall adsorption capacity [74,92,93]. Leveraging nanomaterials (NMs) as adsorbents presents a promising avenue due to their potential for high adsorption efficiency [94,95]. Laboratory testing conducted with specially formulated aqueous solutions (not actual wastewater) has yielded encouraging results, suggesting the viability and interest of this approach. In recent years, researchers have delved into the investigation of synthesized, functionalized, and characterized NMs for the efficient adsorption of rare earth ions (RE^3+^) from aqueous solutions. This exploration extends beyond resource retrieval considerations to encompass the broader context of environmental and human health impacts associated with REEs and wastewater. For example, Iftekhar and co-workers investigated the mechanism of synthesis of graphite(GA)-g-PAM from free radicals using the EPR spin trapping technique and a possible reaction pathway was proposed [96]. The synthesized nanocomposite was utilized for the removal of REEs, Eu, La, Nd, and Sc from aqueous solutions. 

**Figure 8:**
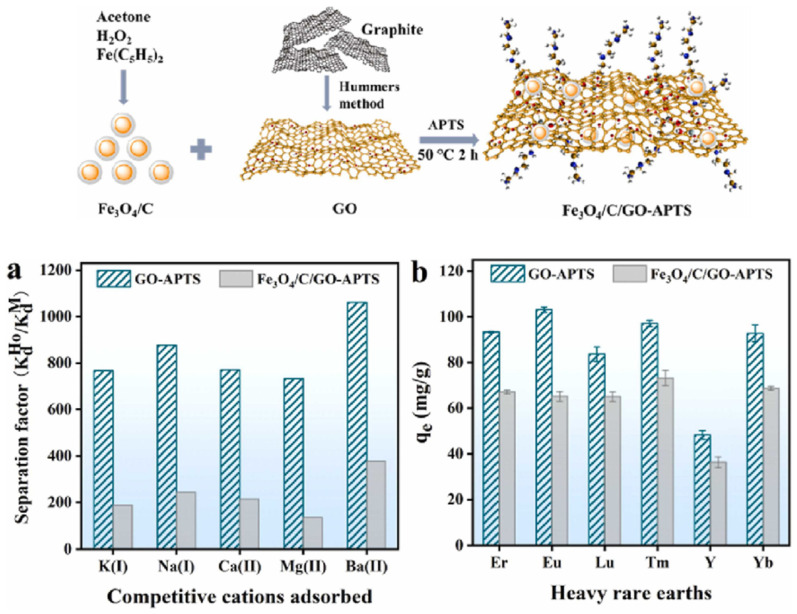
Illustration of grafted magnetic graphene oxide composites preparation. (**a**) The separation factor of coexistent cations in the mixture solution (Experimental conditions: the initial concentration of each cations was about 100 mg/L; adsorbent dosage, 1 g/L; pH, 6.0); (**b**) adsorption capacities of various heavy rare earth elements by GO-APTS and Fe_3_O_4_/C/GO-APTS. Reprinted with the permission from Ref. [97].

Yang and collaborators synthesized amino-grafted magnetic graphene oxide composites through a straightforward one-step cross-linking reaction involving graphene oxide and magnetic Fe_3_O_4_/C nanoparticles, as shown in Figure 8a [97]. The magnetic graphene oxide composites, as synthesized, exhibit remarkable long-term stability when subjected to both acidic and alkaline solutions. These composites display outstanding efficacy in the removal of Ho(III), a prototypical REE, from water. Impressively, their observed adsorption capacity, measured at 72.1 mg of Ho(III) per gram of material, surpasses the capabilities of most previously reported magnetic materials. Even after undergoing 18 adsorption-desorption cycles in acid and alkali solutions, these composites maintain their structural integrity and retain their physicochemical properties. Furthermore, this adsorbent exhibits exceptional adsorption performance, not only for Ho(III) but also for other heavy REEs, including Er(III), Eu(III), Lu(III), Tm(III), Y(III), and Yb(III). 

Figure 8b illustrates the impact of single coexistent ions on Ho(III) adsorption, revealing that the interference of K^+^, Na^+^, Ca^2+^, Mg^2+^, and Ba^2+^ on the removal of Ho(III) can be disregarded. In addition to Ho(III), they also evaluated the adsorption capabilities of graphene oxide-3-aminopropyltriethoxysilane(GO-APTS) and Fe_3_O_4_/C/GO-APTS for other heavy rare earth elements, namely Er(III), Eu(III), Lu(III), Tm(III), Y(III), and Yb(III). It highlights that Ho(III) adsorption remains unaffected by the presence of coexistent ions. The adsorption capacities of GO-APTS for these rare earth elements are provided, ranging from 48.3 mg/g to 103.2 mg/g. In comparison, Fe_3_O_4_/C/GO-APTS exhibits slightly lower adsorption capacities due to its lower APTS content within the composite, as presented in Figure 8c. However, both materials demonstrate significant potential for the extraction of a wide range of rare earth elements.

**Figure 9:**
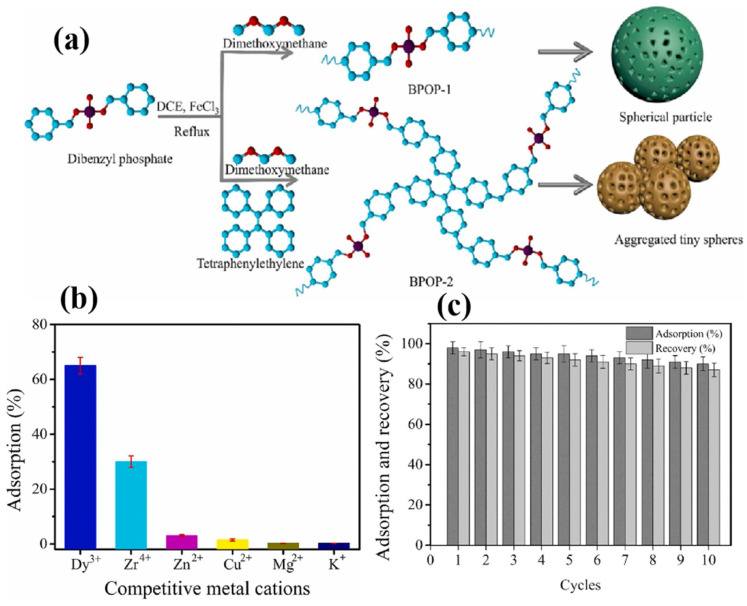
(**a**) Schematic syntheses of phosphate-based porous organic polymers BPOP-1 and BPOP-2. (**b**) Selectivity of Dy^3+^ adsorption to BPOP-2 in the presence of other competitive ions. (**c**) Adsorbent regeneration test to evaluate the adsorption and recovery of Dy^3+^in BPOP-2. Reprinted with the permission from Ref. [99].

In another study, researchers investigated the utilization of ultra-small cerium oxide nanoparticles within a matrix of woven-non-woven polyvinyl alcohol (PVA) nanofibers to create a nanocomposite designed for adsorbing REE ions [98]. Bae and colleagues developed two innovative phosphate-functionalized porous organic polymers, namely BPOP-1 and BPOP-2, as illustrated in Figure 9a. Both of these BPOP materials exhibit exceptional chemical stability across a broad spectrum of pH conditions [99]. The research team assessed the potential of BPOP materials for selectively removing REEs (Eu^3+^, Gd^3+^, Tb^3+^, and Dy^3+^) from aqueous solutions. As illustrated in Figure 9b, BPOP-2 exhibited a remarkable capacity to selectively capture Dy^3+^ ions over other tetravalent, divalent, and monovalent cations. Impressively, nearly all of the adsorbed Dy^3+^ ions were successfully recovered during the initial 10 consecutive adsorption and desorption cycles, as depicted in Figure 9c. This highlights the enduring and robust performance of BPOP-2 over at least 10 cycles.

Polymeric materials have risen as highly promising contenders in the quest for REE recovery, effectively addressing the pressing demand for sustainable and environmentally-friendly techniques. These materials boast a range of advantages, including exceptional selectivity, adaptability in various forms, and the remarkable capacity for regeneration and repeated use. An array of polymeric resins, membranes, and composite materials has been ingeniously developed, showcasing remarkable adsorption capabilities and robust stability in the context of REEs recovery. Furthermore, innovative strategies like redox-copolymers and phosphate-functionalized polymers have emerged, offering exciting prospects for efficient REEs capture and release. Although certain challenges persist, such as enhancing selectivity and optimizing large-scale processes, it is undeniable that polymeric materials play a pivotal role in mitigating environmental hazards and fortifying the security of REEs supplies.

One thing we want to mention here but not discuss in detail for this review paper is the artificial intelligence (AI) used for recovery of REEs. AI offers significant advantages in REE recovery, such as it can analyze vast datasets related to REE resources, mining operations, and processing techniques [100,101,102,103]. By predicting optimal mining sites or suggesting efficient extraction methods to optimize the REE recovery process, AI-powered robots and sorting systems can enhance the recovery of REEs from electronic waste and other recyclables. There are also challenges to overcome. These include the need for high-quality data, the development of specialized AI algorithms, and addressing ethical concerns related to automation and job displacement. However, with ongoing advancements in AI and a growing understanding of its applications, it holds great promise for making REEs recovery more efficient, sustainable, and economically viable.

## 3. Summary

In 2021, China achieved a significant milestone by recovering 27,000 tons of rare earths from production-side waste. This remarkable accomplishment accounted for 16% of the total rare earth oxide production, which amounted to 170,000 tons during that year. Furthermore, both the United States and the European Union have demonstrated a strong commitment to advancing research and development in the field of rare earth recycling technology. Their efforts are accompanied by substantial industrial support. For instance, the European Union’s commitment is exemplified through its substantial investment in the related project. This extensive initiative comprises 19 sub-projects spanning across nine different countries and boasts a total funding amount of EUR 140 million. 

In this paper, we explore the utilization of polymeric materials in the recovery of REEs, which are crucial in various industries. It addresses the challenges arising from the heavy reliance on China for REEs supply and the associated environmental hazards of REEs accumulation in ecosystems. We primarily focus on polymeric materials and their merits in REEs recovery. These materials can be finely tuned for high selectivity, enabling the precise adsorption of specific REEs. The functionalization of polymers enhances their affinity for REEs, thereby enhancing both adsorption and recovery efficiency. Additionally, polymeric materials exhibit versatility, with the ability to be molded into various forms, from beads to membranes, making them adaptable for different scales of operation. Sustainability is a key theme, with an emphasis on the regenerable properties of many polymeric materials used in REEs recovery. This regenerability not only conserves valuable resources but also reduces waste generation. Furthermore, compared to traditional solvent-based extraction methods, polymeric materials operate under milder conditions and generate fewer harmful byproducts.

This review categorizes polymeric materials into distinct groups, including polymeric resins, polymer membranes, cross-linked polymer networks, and nanocomposite polymers. It provides illustrative examples within each category, underscoring their effectiveness in REEs recovery. Case studies demonstrate the impressive adsorption capacities and regenerable nature of these materials. The future direction of polymer materials-based rare earth elements recovery will likely involve a combination of tailored material design, sustainability, economic feasibility, and interdisciplinary collaboration. These efforts aim to address the growing demand for REEs while mitigating environmental impacts and ensuring a stable supply chain for critical industries.

In conclusion, we acknowledge the existing challenges, such as the quest for high selectivity and stability, and the optimization of recovery processes for large-scale applications. Nevertheless, it underscores the promising role of polymeric materials in addressing the urgent need for sustainable and eco-friendly REEs recovery methods. Collaboration between researchers and industry experts continues to drive advancements in these methods, thereby mitigating the environmental and supply chain risks associated with REEs.

## Figures and Tables

**Table 1:** Comparison of different polymeric membranes commonly employed in REE recovery.

Polymeric Membranes	Composition	Advantages	Applilcation
Polymeric Resin Membranes	Cross-linked polymeric resins with functional groups	High REE selectivity, regenerate ability	Solution, hydrometallurgical processes
Polymer Inclusion Membranes (PIMs)	3D-polymer network	Highly versatile, high stability	Continuous membrane-based extraction and separation
Phosphate-Functionalized Polymeric Membranes	membranes incorporate phosphate groups	Strong affinity, Excellent selectivity	Complex solutions, such as acid mine drainage
Amino-Functionalized Polymeric Membranes	membranes contain amino groups	High selectivity	Recovery of specific REEs, wastewater treatment
Magnetic Polymer Membranes	Magnetic nanoparticles embedded within polymer matrices	Easy separation	Selective extraction of specific magnetic REEs
Composite Polymeric Membranes	Combinations of various polymers, fillers, and additives	Customization	Versatile for use in various extraction and separation processes

## Data Availability

Not applicable.

## Abbreviations

REEs	Rare earth elements
DEHDGA	*N*,*N*-di(2-ethylhexyl)-diglycolamide
RE(III)	Rhenium(III)
Al(III)	Aluminum(III)
Fe(III)	Iron(III)
DGA	Diglycolamides
P(FPMAm-co-MAA)	Poly(ferrocenylpropyl methacrylamide-co-methacrylic acid)
Y	Yttrium
Nd	Neodymium
Eu	Europium
Gd	Gadolinium
Dy	Dysprosium
Ce	Cerium
CNT	Carbon nanotube
Fc	Ferrocene
NF	Nanofiltration
UF	Ultrafiltration
PDA	Polydopamine
PVDF	Polyvinylidene difluoride
PTFE	Polytetrafluorethylene
CTA	Cellulose triacetate
PEI	Polyethylenimine
pAAm	Polyacrylamide
PIM	Polymer inclusion membranes
MBAA	*N*,*N*-methylene-bis-acrylamide
PPN	Phosphate polymer nanogel
Kd	Distribution coefficients
NMs	Nanomaterials
GA	Graphite
GO-APTS	Graphene oxide-3-aminopropyltriethoxysilane
PVA	Polyvinyl alcohol
BPOP	Phosphate-functionalized porous organic polymers
AI	Artificial intelligence
